# Different Profile of Serum Leptin between Early Onset and Late Onset Preeclampsia

**DOI:** 10.1155/2014/628476

**Published:** 2014-01-23

**Authors:** Saeedeh Salimi, Farzaneh Farajian-Mashhadi, Anoosh Naghavi, Mojgan Mokhtari, Mahnaz Shahrakipour, Mohsen Saravani, Minoo Yaghmaei

**Affiliations:** ^1^Cellular and Molecular Research Center, Zahedan University of Medical Sciences, Zahedan 9816743175, Iran; ^2^Department of Clinical Biochemistry, School of Medicine, Zahedan University of Medical Sciences, Zahedan 9816743175, Iran; ^3^Department of Pharmacology, School of Medicine, Zahedan University of Medical Sciences, Zahedan 9816743175, Iran; ^4^Department of Medical Genetics, Faculty of Medical Sciences, Tarbiat Modares University, Tehran 14115-111, Iran; ^5^Department of Obstetrics and Gynecology, School of Medicine, Zahedan University of Medical Sciences, Zahedan 9816743175, Iran; ^6^Department of Biostatistics and Epidemiology, School of Public Health, Zahedan University of Medical Sciences, Zahedan 9816743175, Iran; ^7^Pregnancy Health Research Center, Zahedan University of Medical Sciences, Zahedan 9816743175, Iran

## Abstract

*Aim*. This study was designed to clarify the role of leptin and adiponectin in preeclampsia (PE) pathogenesis and different subtypes of preeclampsia. *Method*. This case control study was performed in 45 PE patients and 45 healthy controls matched for age, BMI, and ethnicity. Serum leptin and adiponectin levels were determined by enzyme linked immunosorbent assay (ELISA). *Results*. Maternal serum leptin and adiponectin were significantly higher in PE women than controls. Serum leptin was elevated in early onset preeclampsia (EOPE) and late onset preeclampsia (LOPE) compared to controls. Among PE patients, serum leptin was higher in EOPE than LOPE women. However, serum adiponectin was not different between EOPE and LOPE women. The serum leptin was significantly higher in severe PE than mild PE. The serum adiponectin was significantly elevated in severe PE compared to controls. Significant positive correlation was observed between leptin and adiponectin and also between leptin and BMI in controls. Moreover significant positive correlation was observed between adiponectin and BMI in PE patients and controls. *Conclusion*. The present study showed that serum leptin level may play a significant role as a biomarker to differentiate early and late onset PE and also its relation to BMI and severity of disease.

## 1. Introduction

Preeclampsia (PE) is a complication of pregnancy which is characterized by hypertension and proteinuria. It affects 2% to 5% of pregnancies and is a major contributor to fetal, neonatal, and maternal morbidity and mortality. PE symptoms might be revealed from 20 weeks of gestation up to six weeks postpartum and is considered early onset before 34 weeks of gestation [1]. Although PE has an unknown etiology, metabolic, placental, genetic, and immune factors have been concerned in its etiopathogenesis [2].

There is convincing evidence for the association between obesity related complications and preeclampsia [3]; however, the mechanism by which excess adipose tissue causes developing PE in pregnant women remains unknown. Adipose tissue is not only involved in energy storage but can also act as an endocrine tissue producing a wide range of cytokines and chemokines such as adiponectin and leptin. It is shown that these two adipokines play a role in normal pregnancy, as well as in complications of pregnancy, including PE [4].

Leptin is a 16 kDa protein hormone which plays an important role in modulating satiety, energy homeostasis, and reproductive biology. There are several studies showing that leptin serum concentration increases during pregnancy [4–7]. However some studies demonstrate decreased or unchanged circulating leptin concentrations in PE patients [8, 9].

Adiponectin is a 30 kDa protein which is synthesized by adipocytes and considered as an anti-inflammatory, insulin-sensitizing, and antiatherogenic adipokine [10]. Plasma adiponectin levels are affected by multiple factors including gender, age, and lifestyle [11]. During the course of pregnancy, maternal adiponectin secretion gradually declines [12]. In PE, a paradoxical and significant increase of circulating adiponectin of 30% to 87% has been described in most [6, 13–15] but not all studies [16, 17].

PE could be characterized into 2 different disease entities: early-onset PE (EOPE) and late-onset PE (LOPE) which is associated with different fetal and maternal effects, heritability, biochemical markers, and clinical symptoms [18]. Furthermore EOPE and LOPE might have different pathogenesis [17]. There are several studies which showed different leptin and adiponectin profiles between EOPE and LOPE [7, 17, 19, 20].

Considering these variations, the present study was designed to investigate the role of leptin and adiponectin in EOPE and LOPE and their relations to BMI and PE severity, in the southeastern Iranian population.

## 2. Materials and Methods

This case control study was undertaken in 45 normal pregnant women and 45 patients with PE. Written informed consent was obtained from all participants after approval by the Zahedan University of Medical Sciences Ethics Committee.

Preeclampsia was defined as the presence of hypertension (systolic blood pressure ≥140 mm Hg or diastolic blood pressure ≥90 mm Hg on at least two occasions, 4 h to 1 week apart) and proteinuria (≥300 mg in a 24 h urine collection or at least one dipstick measurement ≥1+) [21]. Blood pressure was taken from the patient in an upright position, after a 10-minute rest period with a mercury sphygmomanometer. The right arm was used for the measurement and it was placed in a horizontal position at heart level [22]. The BMI was calculated according to the formula: weight (kg)/height (m^2^). Exclusion criteria included twin or multiple pregnancies or any evidence of previous medical disease. 45 normal pregnant volunteers were randomly enlisted from the Obstetrics Department of Ali-Ebne-Abitaleb Hospital that did not have any evidence of previous medical illness.

None of PE patients and controls received any medications before blood sampling. To obtain and clarify serum, the samples were left to stand at room temperature for at least 30 min to allow the blood to clot, centrifuged at 2000 g for 15 min and then the serum was removed into separate aliquots. All samples were cooled to −80°C and stored at that temperature until assays were performed.

Serum concentrations of leptin and adiponectin were determined by enzyme-linked immunosorbent assay (ELISA,). The assays were conducted according to manufacturer's protocols (R&D Systems Inc. Minneapolis, MN, USA).

### 2.1. Statistical Analysis

Statistical analysis was performed using SPSS 15.0 software. Quantitative values were presented as mean ± SD and based on normality of leptin levels (Kolmogorov-Smirnov test), the differences between two groups were examined by independent Student's *t*-test. Due to nonnormality of adiponectin levels, Mann-Whitney test was used to compare this parameter between two groups. Fisher's exact test has been used for comparison of categorical variables. The correlation was analyzed by Pearson correlation coefficients. Box plot graphs were used to show leptin and adiponectin values in different groups. For all tests performed, a two-sided *P* value of less than 0.05 was considered as significant.

## 3. Results

The clinical and demographic characteristics of subjects are shown in Table 1.

As shown in Table 1, there were no significant differences in age, birth weight, primiparity, and BMI between PE and control groups. Systolic and diastolic pressures were significantly higher in the PE group compared to control group (*P* = 0.0001). Although the gestational age was lower in PE patients than controls (*P* = 0.02), there was no statistical difference in gestational age between EOPE with LOPE and severe with mild PE.

The serum leptin levels in PE patients were 35.7 ± 16.4 ng/mL and in the control group it was 22.6 ± 7.7 ng/mL which shows a significant elevation in PE patients (*P* < 0.001). The serum adiponectin levels in PE patients were 16.6 ± 3.9 *μ*g/mL whereas in the control group it was 14.8 ± 4.6 *μ*g/mL which also indicates a significant elevation in PE patients (*P* = 0.038). Furthermore there were no significant differences in serum leptin/adiponectin ratio in PE and control pregnant women (*P* < 0.05).

**Figure 1:**
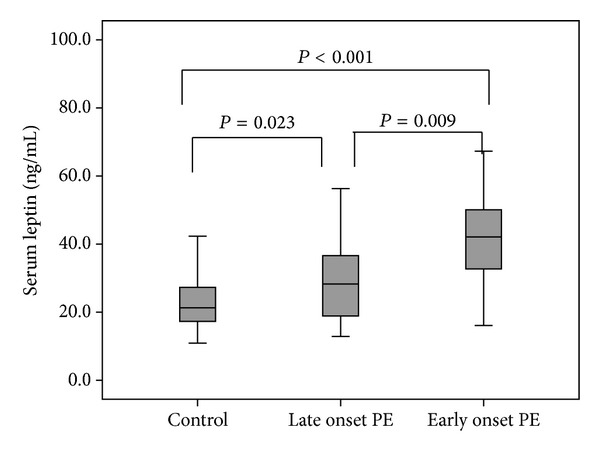
Comparison of the median serum leptin levels between early onset PE, late onset PE, and controls.

**Figure 2:**
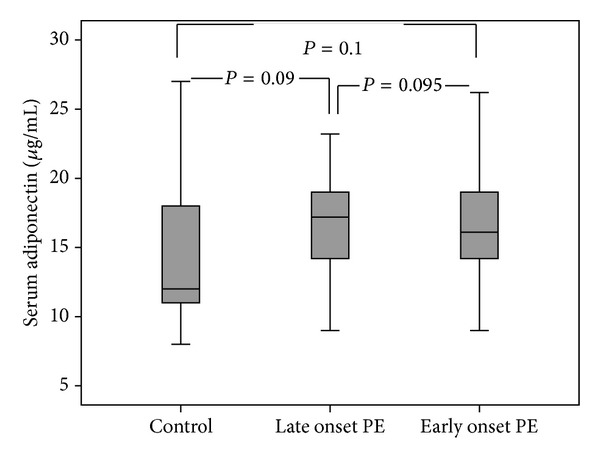
Comparison of the median serum adiponectin levels between early onset PE, late onset PE, and controls.

From 45 PE patients, 20 individuals had early onset and 25 had late onset PE. EOPE is usually defined as PE that develops before 34 weeks of gestation, whereas LOPE develops at or after 34 weeks of gestation. The serum leptin levels in EOPE were 42.7 ± 16.2 ng/mL whereas in LOPE were 30.1 ± 14.6 ng/mL which was significantly different (*P* = 0.009). Moreover the serum leptin levels in EOPE and LOPE patients were significantly higher than controls (*P* < 0.001 and *P* = 0.023, resp.) (Figure 1). The serum adiponectin levels were not statistically different between EOPE and LOPE (16.8 ± 4.2 *μ*g/mL versus 16.5 ± 3.8 *μ*g/mL, *P* = 0.095). The adiponectin levels in EOPE and LOPE were not significantly higher than controls (*P* = 0.09 and *P* = 0.1, resp.) (Figure 2).

**Figure 3:**
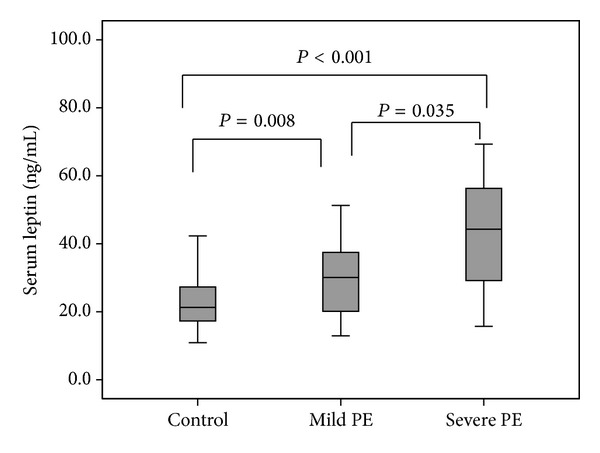
Comparison of the median serum leptin levels between severe PE, mild PE, and controls.

**Figure 4:**
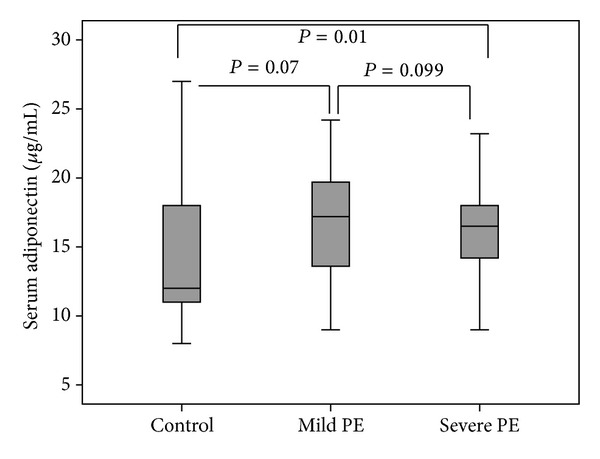
Comparison of the median serum adiponectin levels between severe PE, mild PE, and controls.

Among 45 preeclamptic patients, 18 individuals had severe and 27 individuals had mild preeclampsia. The serum leptin levels in “severe PE” group were significantly higher than “mild PE” group (42.2 ± 16.7 ng/mL versus 31.5 ± 15.1 ng/mL, *P* = 0.035). In addition the serum leptin in “severe PE” and “mild PE” groups was significantly higher than controls (*P* < 0.001 and *P* = 0.008, resp.) (Figure 3). However, the differences between serum adiponectin levels in severe and mild PE groups were not significant (17.06 ± 4 and 17.7 ± 3.9 *μ*g/mL, *P* = 0.099); however, the adiponectin levels were significantly higher in severe PE patients compared to controls (*P* = 0.01) (Figure 4).

**Figure 5:**
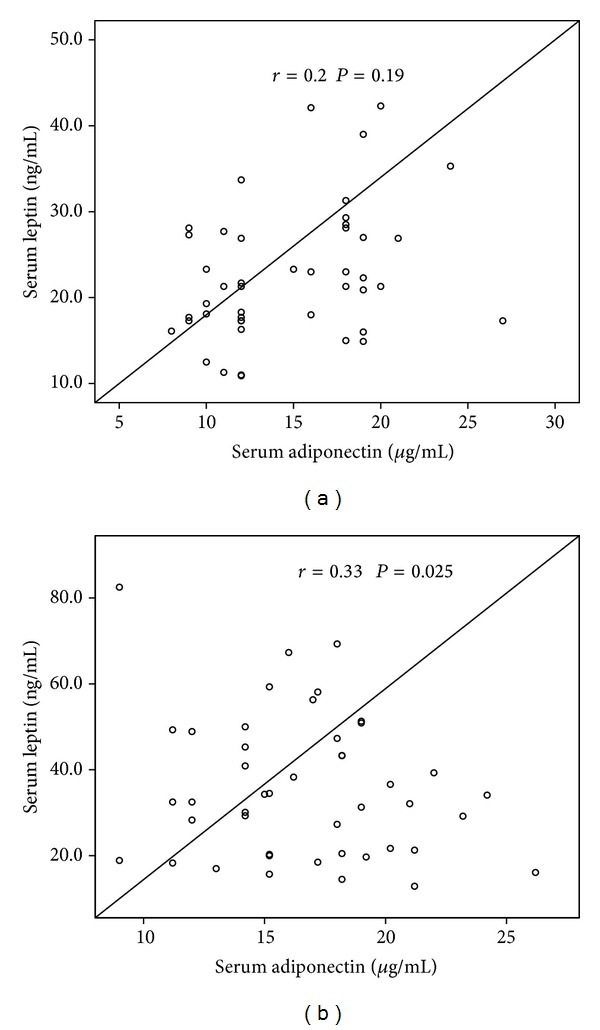
Correlation between serum leptin and adiponectin levels in (a) preeclamptic women and (b) healthy controls.

**Figure 6:**
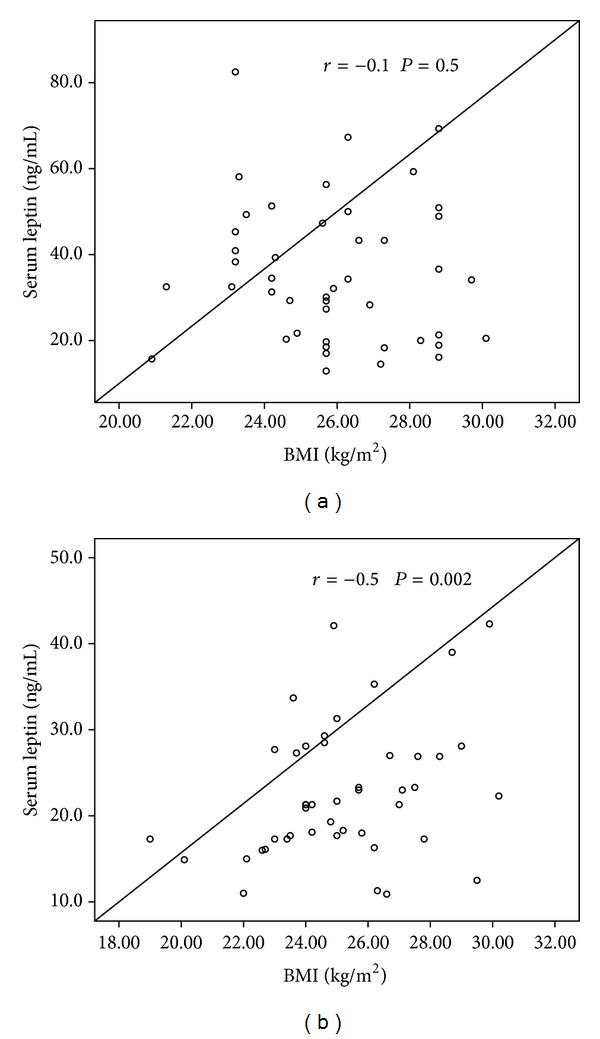
Correlation between serum leptin levels and BMI in (a) preeclamptic women and (b) healthy controls.

**Figure 7:**
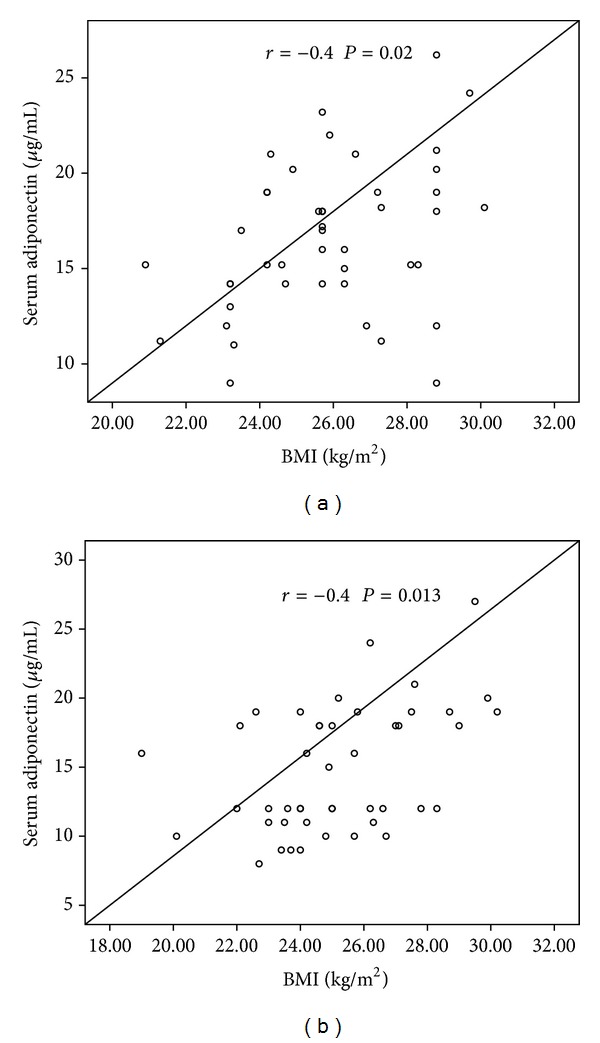
Correlation between serum adiponectin levels and BMI in (a) preeclamptic women and (b) healthy controls.

There was no correlation between serum adiponectin and leptin levels in the PE group (Figure 5(a)), whereas in the control group there was a significant correlation between these adipocyte hormones (*r* = 0.33, *P* = 0.025) (Figure 5(b)). In addition no correlation was found between BMI and leptin levels in the PE group (*r* = −0.1, *P* = 0.5) (Figure 6(a)), whereas a significant correlation was observed in the control group (*r* = 0.5, *P* = 0.002) (Figure 6(b)). A significant correlation was observed between BMI and adiponectin in PE (*r* = 0.4, *P* = 0.02) (Figure 7(a)) and control pregnant women (*r* = 0.4, *P* = 0.013) (Figure 7(b)).

## 4. Discussion

The results of the current investigation revealed a significant elevation of leptin and adiponectin in pregnant women with PE compared with normotensive pregnant women which is in accordance with many other reports [4–7]. However, some studies showed decreased or unchanged circulating leptin [8, 9] or adiponectin [16, 17] levels in PE patients.

Other reports have revealed that leptin protein and mRNA levels were increased in placentas from PE women as compared to healthy pregnant women [23–25]. Furthermore, increased levels of circulating leptin in PE [4–7] even before the clinical onset of the disease [26–29] have been shown in most of the studies and it seems that higher leptin levels have a prognostic role for the development of PE. As described by Ning et al., each 10 ng/mL increase in leptin concentration is associated with a 30% increase in PE risk [28]. Another study indicated that hypoxia upregulates placental leptin gene expression through a transcriptional mechanism involving distinct sequences on the promoter [30]. Ramsay et al. [15] reported an elevation of plasma adiponectin concentration in women with PE compared with controls and suggested that adiponectin released during pregnancy could be a physiological response to minimize fat accumulation and also to decrease endothelial damage. However, Liu et al. believed that circulating adiponectin levels in PE women might be due to a reduced degradation/elimination rather than an increased synthesis of this adipokine [31].

Another remarkable observation of this study was significant increased serum leptin levels in early onset PE compared to late onset PE. However there was no significant difference in adiponectin level between these two PE subtypes. This finding was in line with Masuyama et al.'s investigation in Japanese women. They indicated the significant elevation of leptin in EOPE and LOPE compared to controls; however, adiponectin was increased only in LOPE [7]. Furthermore, in support of the present study Hogg et al. reported that maternal leptin concentration was elevated in EOPE patients. They believed that hypomethylation of LEP gene in placenta may lead to increased overall LEP expression [19].

Despite the current results, Molvarec et al. found no statistically significant difference in serum leptin concentration between late and early onset PE and among PE patients with mild and severe form of the disease. Moreover they indicated that serum leptin levels were not associated with BMI in PE patients [20].

Furthermore we observed that elevated level of leptin in PE patients has direct relation with severity of this disease. However, there was no association between adiponectin and PE severity.

Similar to the results of the present study, Abd-Alaleem et al. reported that serum adiponectin level in preeclamptic women was significantly higher than normal pregnant women especially in cases of severe preeclampsia [32]. Moreover Nien et al. reported higher adiponectin in severe preeclampsia [33]. In contrast, Dalamaga et al. observed not only no significant differences in adiponectin and leptin levels between preeclamptic and control pregnant women but also no differences between mild and severe PE among preeclamptic women [34]. Another study revealed that severe PE was associated with higher median plasma concentration of adiponectin than that of normal pregnant women. They suggested that it could be a result of a compensatory mechanism to the metabolically altered, proatherogenic and antiangiogenic condition of severe PE [33].

In the current study, it was also revealed that serum adiponectin correlated positively with serum leptin in healthy pregnant women but not in PE patients. In contrast, Nakatsukasa et al. found a significant negative correlation between leptin and adiponectin in healthy pregnancies but not in PE patients [35]. Khosrowbeygi and Ahmadvand observed that serum levels of adiponectin were significantly higher in the preeclamptic patients compared to controls and serum adiponectin had a significant negative correlation with body mass index in PE patients but not in normal pregnant women [36]. In another study Herse et al. reported elevated adiponectin levels in normal pregnancy and correlated this with prepregnancy body mass index, whereas plasma leptin levels decreased in normal pregnancy [37].

Our data shows that there was correlation between leptin and BMI in healthy pregnant women but not in PE patients. Moreover, our results clarified a significant positive correlation between BMI and adiponectin in both control and PE groups. The results of different studies in this field are inconsistent. For instance, Hendler et al. reported that women with severe PE and BMI ≥25 kg/m^2^ have decreased adiponectin and increased leptin levels, whereas normal weight women with PE have increased adiponectin levels [6]. However, Mendieta Zeron et al. observed that obese women (BMI ≥40 kg/m^2^) had significantly higher level of serum leptin (*P* < 0.01) and the value of 40 ng/mL of this hormone appears to be predictive in developing PE [38]. Furthermore Suwaki et al. demonstrated that overweight patients with PE showed significantly lower adiponectin levels [39].

There is strong evidence that placenta, rather than maternal adipose tissue, is responsible for the rise in maternal leptin concentrations during pregnancy [40]. This feedback response to significantly elevated leptin levels may increase nutrient delivery to the underperfused placenta in PE [40–43].

These paradoxical data could be due to factors including different criteria for diagnosis of PE, medications that affect energy balance, gestational age, smoking, and ethnicity of cases. Nevertheless, most studies describe upregulation of leptin in PE [44].

In summary, this study demonstrated that leptin and adiponectin levels were increased in PE patients and only the serum leptin was significantly higher in EOPE compared to LOPE. Also the leptin level was different between severe and mild PE. We found positive correlation between leptin with adiponectin and leptin with BMI in control group. The findings of current study are consistent with the role of adipocytes hormones, in the pathophysiology of PE, and have important diagnostic and therapeutic implications.

## Figures and Tables

**Table 1:** Clinical and demographic characteristics of PE patients and controls.

Variable	PE (*n* = 45)	Control (*n* = 45)	*P* value
Age (years)	27.9 ± 6.7	26.7 ± 7.2	0.4
Gestational age (days)	251.6 ± 27.6	265 ± 19.3	0.02
Birth weight (g)	2675 ± 935	2794 ± 596	0.5
Diastolic blood pressure (mm Hg)	155.6 ± 16.6	112 ± 12.3	<0.001
Systolic blood pressure (mm Hg)	97.2 ± 9.3	69.2 ± 9.6	<0.001
Primiparity, *n* (%)	12 (27)	20 (44)	0.06
BMI (kg/m^2^)	25.9 ± 3	25.3 ± 2.5	0.3
Family history of preeclampsia *n* (%)	14 (31)	22 (49)	0.07
